# Reducing the Risk of Unintentional Doping From Supplements: A Practical Guide for Athletes and Support Teams

**DOI:** 10.1155/tsm2/5919439

**Published:** 2026-08-23

**Authors:** Laura Mancin, Graeme L. Close, Asker Jeukendrup, Marco Freschi, Jamie Pugh, Massimiliano Sala, Veronica Baioccato, Marco Vecchiato, Giuseppe D’Antona, Stefano Palermi

**Affiliations:** ^1^ Research Institute for Sport and Exercise Sciences, Liverpool John Moores University, Liverpool, UK, ljmu.ac.uk; ^2^ Loughborough University, Loughborough, UK, lboro.ac.uk; ^3^ Juventus Football Club, Turin, Italy; ^4^ Manchester City Football Club, Manchester, UK; ^5^ Department of Medicine, Sports and Exercise Medicine Division, University of Padova, Via Giustiniani 2, Padova 35128, Italy, unipd.it; ^6^ Department of Public Health, Experimental and Forensic Medicine, University of Pavia, Pavia 27100, Italy, unipv.eu; ^7^ Department of Medicine and Surgery, UniCamillus Saint Camillus International University of Health Sciences, Rome 00187, Italy

**Keywords:** antidoping, athlete safety, decision-making, dietary supplements, inadvertent doping, sports nutrition, supplement contamination

## Abstract

**Context:**

Dietary supplements are widely used across all levels of sport. While the ‘food first but not food only’ approach remains central to sports nutrition, selected supplements may support performance, recovery or health when used appropriately. However, variable regulation, product contamination, mislabelling and inappropriate decision‐making may expose athletes to inadvertent antidoping rule violations (ADRVs).

**Evidence Acquisition:**

This structured narrative review searched PubMed and Scopus for literature published between January 2000 and December 2025. Evidence from primary studies, randomised controlled trials, systematic reviews, meta‐analyses, mechanistic studies, contamination analyses, behavioural research, consensus statements and official guidance documents from major sports and antidoping organisations was critically evaluated and synthesised. A PRISMA‐style literature identification and evidence‐selection diagram is provided as Supporting Table S1; the review was not designed as a systematic review or meta‐analysis.

**Study Design:**

Narrative clinical review.

**Level of Evidence:**

Level V for the overall narrative framework, with supplement‐specific strength of recommendation reported separately.

**Results:**

Evidence supporting supplementation varies substantially across categories. Creatine and caffeine have the most consistent evidence for selected performance outcomes, whereas beta‐alanine, dietary nitrate and sodium bicarbonate show context‐specific benefits. Iron and vitamin D have clearer clinical indications when deficiency is present, but limited evidence supports routine use in replete athletes. Many recovery‐ or health‐oriented supplements rely on smaller, heterogeneous studies or indirect outcomes. Contamination risk is also heterogeneous and is highest for multi‐ingredient products, preworkout supplements, weight‐loss products, muscle‐building products, ‘testosterone boosters’, selective androgen receptor modulators (SARMs) and other substances positioned as supplements despite pharmacological or prohibited profiles. Effective risk reduction requires individual nutritional assessment, evidence‐based selection, third‐party certification, product verification, education of athletes and Athlete Support Personnel, and systematic documentation.

**Conclusions:**

Supplement use in athletes should be considered a controlled, individualised and documented intervention rather than routine practice. The proposed framework integrates efficacy, evidence quality, mechanisms of action, contamination pathways, behavioural determinants, strict liability and antidoping risk to support safer supplementation decisions. Protecting athlete health and the integrity of clean sport requires a shift from performance‐driven to risk‐aware supplementation.

Strength‐of‐Recommendation Taxonomy (SORT): Overall framework: B/C, based on consensus statements, position stands, limited controlled studies and expert‐informed synthesis. Supplement‐specific ratings are provided in Table 2.

## 1. Introduction

The pursuit of peak performance in sport requires continuous optimisation of training, recovery, nutrition and health. In many athletic contexts, habitual dietary intake may not fully meet the energetic, macronutrient or micronutrient demands imposed by repeated training sessions, travel, competition schedules, extreme environments or limited access to appropriate food choices [1–3]. Consequently, dietary supplements are widely used by athletes, with prevalence estimates commonly ranging from approximately 40% to 70%, depending on sport, competitive level, sex, age and assessment methodology [4, 5].

This widespread use creates a central paradox in contemporary sports medicine. Some supplements are among the most evidence‐based and practically useful interventions available to athletes, whereas the same market may expose athletes to substances that are ineffective, mislabelled, contaminated or prohibited under antidoping regulations [6–9]. Thus, the clinical question is not simply whether a supplement can improve performance, but whether its expected benefit justifies its safety, legal and antidoping risk in a specific athlete and context.

Dietary supplements may be used to correct nutrient deficiencies, provide convenient sources of energy or protein, support hydration or target specific physiological mechanisms related to performance, recovery or adaptation [6–10]. Consensus statements, including those from the International Olympic Committee (IOC), support the targeted use of a limited number of supplements in well‐defined scenarios when decisions are made by qualified professionals and grounded in evidence‐based practice [6]. However, the commercial supplement landscape is substantially broader than the evidence base. Many products are marketed with performance, recovery, or body‐composition claims that exceed available scientific support.

Unlike pharmaceutical products, dietary supplements are generally regulated as foods or food‐adjacent products in many jurisdictions and premarket demonstration of efficacy and safety is often not required. In the United States, the Dietary Supplement Health and Education Act of 1994 shifted substantial responsibility for safety and labelling to manufacturers rather than regulators [11]. Similar regulatory asymmetries occur internationally, creating variability in quality control, ingredient verification and postmarket surveillance. Independent analyses have repeatedly identified mislabelling and contamination, with undeclared anabolic‐androgenic steroids, stimulants, selective androgen receptor modulators (SARMs), peptide‐related compounds and other pharmacologically active substances reported in commercially available products [12–26].

From an antidoping perspective, these risks are amplified by the principle of strict liability. Under the World Anti‐Doping Code, athletes are responsible for any prohibited substance found in their samples, regardless of intent or source [12, 13]. Therefore, ingestion of a contaminated supplement may result in an adverse analytical finding (AAF) and potentially an antidoping rule violation (ADRV), with consequences that may include disqualification, suspension, financial loss, reputational damage and psychological distress. Athlete Support Personnel (ASP), including physicians, dietitians, coaches and trainers, may also be implicated when they recommend, provide or facilitate the use of high‐risk products.

The available evidence on supplement‐related ADRVs remains heterogeneous. Data derive from laboratory analyses of selected products, retrospective case investigations, cross‐sectional surveys, antidoping reports, behavioural studies and clinical or performance trials. Prospective studies linking real‐world supplement exposure, contamination risk and athlete behaviour are scarce. Therefore, a practical review must integrate several evidence streams: sports nutrition efficacy, mechanisms of action, contamination pathways, antidoping regulation, behavioural determinants and implementation strategies.

The aim of this structured narrative review is to provide a practical, clinically oriented framework to reduce the risk of unintentional doping from supplement use. We critically examine the evidence supporting common supplements, mechanisms of action, contamination pathways, behavioural drivers of misuse, strength of recommendations and risk‐management strategies. By integrating sports nutrition, antidoping science and behavioural medicine, we propose a pragmatic approach for athletes and support teams that preserves the potential benefits of targeted supplementation while reducing preventable risk.

## 2. Methods and Evidence Selection

### 2.1. Review Design and Rationale

This manuscript was designed as a structured narrative review with a translational and practical focus. It was not designed as a systematic review, scoping review or meta‐analysis. Accordingly, formal PRISMA or PRISMA‐ScR methodology was not used for quantitative screening, duplicate removal, risk‐of‐bias scoring or pooled effect estimation. Nevertheless, in response to editorial feedback and to improve transparency, we provide a PRISMA‐style literature identification and evidence‐selection diagram in Supporting Table S1. This diagram describes how evidence domains were identified, screened for relevance and incorporated into the narrative synthesis; it should not be interpreted as a formal systematic‐review flowchart.

The narrative approach was selected because the objective was not to estimate a single intervention effect but to integrate heterogeneous evidence relevant to real‐world decision‐making: supplement efficacy, biochemical mechanisms, contamination data, antidoping regulations, athlete behaviour, product certification and practical risk management.

### 2.2. Search Strategy

Electronic searches were performed in PubMed and Scopus for publications from January 2000 to December 2025. Search terms included combinations of ‘dietary supplements’, ‘sports supplements’, ‘athletes’, ‘sports nutrition’, ‘ergogenic aids’, ‘creatine’, ‘caffeine’, ‘beta‐alanine’, ‘dietary nitrate’, ‘sodium bicarbonate’, ‘protein’, ‘iron’, ‘vitamin D’, ‘doping’, ‘anti‐doping’, ‘adverse analytical finding’, ‘anti‐doping rule violation’, ‘contamination’, ‘adulteration’, ‘mislabeling’, ‘SARMs’, ‘stimulants’, ‘third‐party certification’, ‘supplement safety’, ‘decision‐making’, ‘risk perception’, ‘athlete behaviour’ and ‘Athlete Support Personnel’. Reference lists of relevant articles, consensus statements and position stands were manually screened to identify additional landmark or mechanistic publications.

Particular attention was given to literature with direct implications for clinical practice, elite sport, athlete education, antidoping compliance and decision‐making by ASP.

### 2.3. Eligibility Criteria and Evidence Prioritisation

Eligible publications addressed at least one of the following domains: supplement prevalence in athletes; efficacy and safety of sports supplements; mechanisms of action; contamination and mislabelling; supplement‐related AAFs or ADRVs; behavioural determinants of supplement use; risk‐management strategies; certification systems; and official antidoping guidance. Priority was given to systematic reviews, meta‐analyses, randomised controlled trials, prospective studies, laboratory contamination studies, high‐quality narrative reviews, consensus statements, position stands and official documents from recognised sport, nutrition and antidoping organisations. Animal‐only studies, conference abstracts, non‐English papers and articles without sufficient methodological detail were not prioritised.

Guidance documents from the IOC, World Anti‐Doping Agency (WADA), Australian Institute of Sport (AIS), American College of Sports Medicine and International Society of Sports Nutrition were used because they represent widely implemented frameworks in sport practice. These sources were not treated as definitive evidence in isolation; they were interpreted alongside primary research, systematic reviews and contamination studies.

### 2.4. Evidence Interpretation

Evidence was interpreted by considering study design, methodological rigour, consistency of findings, magnitude and practical relevance of observed effects, biological plausibility, applicability to athlete populations, safety profile, product quality and antidoping implications. Potential biases considered included industry funding, publication bias, short intervention duration, small sample sizes, male‐dominant or nonelite cohorts, inconsistent dosing strategies, reliance on surrogate outcomes and limited real‐world surveillance data.

Because the reviewed literature is heterogeneous, quantitative synthesis was not appropriate. Instead, we provide a critical narrative synthesis and supplement‐specific strength‐of‐recommendation ratings to support practical decision‐making.

## 3. Critical Appraisal of the Evidence

The evidence‐based supporting sports supplementation is uneven. A small group of supplements, particularly creatine and caffeine, has been evaluated across numerous controlled trials and reviews, with generally consistent benefits for selected performance outcomes [8–10, 27–30]. In contrast, several recovery‐oriented or health‐oriented products are supported by mechanistic rationale, small trials or indirect outcomes rather than consistent performance data [31–36]. Therefore, recommendations should distinguish between supplements with reproducible performance effects, supplements with clinical indications when deficiency exists and supplements with limited evidence for routine use.

Contamination evidence also varies by product category and study design. Large analytical surveys have reported contamination with undeclared anabolic agents or stimulants, but sampling is often opportunistic and may over‐represent higher risk products or online sources [12–23]. Conversely, underreporting is likely in real‐world ADRV investigations because product samples may no longer be available, batch‐level testing may not be performed, and athletes may not disclose all products used. Thus, the true population‐level contribution of contaminated supplements to ADRVs remains difficult to quantify. The most defensible interpretation is that contamination risk is real, nonuniform and clinically relevant, especially for multi‐ingredient products marketed for muscle gain, weight loss, preworkout stimulation or hormonal modulation.

Behavioural evidence further complicates interpretation. Athletes do not choose supplements solely on the basis of nutritional need or scientific evidence. Peer influence, coach recommendations, perceived performance pressure, social media marketing, sponsorship, body‐image concerns and misperceptions that ‘natural’ or legally sold products are safe all influence use [37–41]. Therefore, unintentional doping prevention cannot rely exclusively on analytical testing or product certification; it also requires education, risk perception, documentation and organisational culture change.

Table 1 summarises the critical appraisal of key evidence domains and how they translate into practice.

**TABLE 1:** Critical appraisal of the principal evidence domains relevant to dietary supplement use in athletes.

Evidence domain	Main findings	Limitations/contradictions	Practical implication
Supplement efficacy	Creatine and caffeine have the most consistent evidence; beta‐alanine, nitrate and bicarbonate are context‐specific; many recovery supplements remain emerging.	Heterogeneous designs, small samples, short interventions, sport‐specific variability and responder/nonresponder effects.	Use supplement‐specific recommendations rather than broad class‐level claims.
Clinical supplementation	Iron and vitamin D have stronger rationale when deficiency or insufficiency is present.	Performance benefit in replete athletes is uncertain; unnecessary use can create harm or distraction.	Require biochemical or clinical indication before prescribing.
Contamination studies	Laboratory surveys show undeclared anabolic agents, stimulants, SARMs and other compounds in some products.	Sampling may be biased towards high‐risk products; true exposure prevalence is uncertain.	Treat risk as product‐category dependent; avoid high‐risk categories.
Certification systems	Third‐party testing reduces but does not eliminate risk.	Batch coverage, logo misuse and supply‐chain changes remain possible.	Verify certification status and batch number before use.
Behavioural science	Risk perception, social influence and marketing contribute to misuse.	Few intervention studies test education programmes against hard antidoping outcomes.	Combine education with organisational policies and documentation.
Antidoping regulation	Strict liability makes athletes responsible for substances detected in their samples.	Intent and source may affect sanction severity but not necessarily the existence of an AAF.	Treat supplementation as a controlled clinical decision.

*Note:* Summary of the main findings, methodological limitations, conflicting evidence and practical implications for clinical decision‐making and antidoping risk management. The table highlights the heterogeneity of the available evidence and supports a risk‐aware interpretation of supplement use rather than a purely efficacy‐based approach.

## 4. The Role, Mechanisms and Benefits of Supplements

Dietary supplements encompass a heterogeneous group of products, including sports foods, fortified foods, isolated nutrients and nonfood bioactive compounds. We adopt the IOC definition of supplements as substances intentionally consumed in addition to the habitual diet to achieve a specific health or performance benefit [6]. From a practical perspective, supplements can be grouped into (i) products used to correct or prevent deficiencies, (ii) sports foods that provide convenient energy, protein, fluid or electrolytes and (iii) performance supplements targeting specific mechanisms such as phosphocreatine availability, central nervous system stimulation, buffering capacity or nitric oxide bioavailability [6–10].

Importantly, mechanism is not equivalent to recommendation. A supplement may produce a measurable biochemical effect without improving athlete‐relevant outcomes. Conversely, a supplement with clear efficacy in one sport or physiological context may be irrelevant or even counterproductive in another. Practical decisions should therefore consider mechanism, magnitude of benefit, evidence consistency, safety, individual response, product quality and antidoping implications.

### 4.1. Supplements With Relatively Strong Performance Evidence

Creatine monohydrate increases intramuscular creatine and phosphocreatine stores, supporting rapid ATP resynthesis during repeated short‐duration, high‐intensity efforts. Evidence is strongest for repeated sprint ability, strength, power, lean mass accretion during resistance training and high‐intensity training capacity [27–29]. The most clinically relevant limitation is context dependence: benefits are smaller or absent when phosphocreatine availability is not limiting, and increased body mass may be undesirable in some endurance or weight‐category settings. From an antidoping perspective, pure certified creatine monohydrate has low intrinsic ADRV risk, whereas multi‐ingredient creatine‐containing products may carry higher contamination risk.

Caffeine primarily acts as an adenosine receptor antagonist, increasing alertness, reducing perceived exertion and modulating neuromuscular function. It has broad evidence for endurance performance, vigilance, reaction time and selected strength or power tasks [30, 42]. However, inter‐individual variability is substantial and may relate to habitual intake, sleep status, anxiety, gastrointestinal tolerance, timing, dose and genetic factors. The practical recommendation is therefore to test caffeine in training before competition, avoid excessive doses and avoid unfamiliar preworkout blends that may contain undeclared stimulants.

Beta‐alanine increases muscle carnosine, improving intracellular buffering during high‐intensity exercise characterised by hydrogen‐ion accumulation. Benefits are most plausible for efforts of approximately 1–4 min and repeated high‐intensity exercise but require chronic loading and are not universal [43–45]. Dietary nitrate, commonly provided as beetroot juice or nitrate‐rich foods, increases nitric oxide bioavailability through the nitrate–nitrite–NO pathway and may improve exercise efficiency or performance in selected endurance and intermittent contexts [46, 47]. Sodium bicarbonate increases extracellular buffering capacity and may improve repeated high‐intensity efforts, but gastrointestinal symptoms often limit usefulness [48, 49]. For these supplements, the evidence is best described as moderate and sport‐specific rather than universally strong.

### 4.2. Supplements With Primarily Clinical or Nutritional Indications

Carbohydrate products, electrolytes and protein supplements are often best conceptualised as sports foods rather than pharmacological ergogenic aids. Their value lies in convenience and feasibility when food‐based strategies cannot meet competition, recovery, or travel demands [7–10, 50, 51]. Protein supplementation can support muscle protein synthesis and adaptation when total dietary protein intake is insufficient or timing constraints make whole‐food intake impractical but provides limited additional benefit when daily protein intake and distribution are already adequate [51, 52].

Iron and vitamin D illustrate the difference between clinical correction and indiscriminate performance enhancement. Iron supplementation is appropriate when deficiency or low iron availability is documented, particularly in endurance athletes, female athletes and those with high losses or restricted diets; however, routine supplementation in iron‐replete athletes is inappropriate and may cause adverse effects [53]. Vitamin D supplementation may correct deficiency and support bone, muscle and immune health, but performance benefits in vitamin‐D‐sufficient athletes remain inconsistent [54, 55]. These examples reinforce the need for biochemical assessment rather than routine use.

### 4.3. Supplements With Emerging or Limited Evidence

Collagen or gelatin with vitamin C has a plausible mechanistic role in connective tissue remodelling when combined with appropriate mechanical loading, but clinical evidence for injury prevention or return‐to‐play outcomes remains emerging [31]. Curcumin, polyphenols and omega‐3 fatty acids may influence inflammatory or oxidative pathways, but performance and recovery effects are heterogeneous, and product formulations vary substantially [32, 33]. Probiotics may support gastrointestinal or immune outcomes in selected contexts, but effects are strain‐specific and cannot be generalised across products [34]. Menthol may alter thermal perception during exercise in the heat but should not replace evidence‐based cooling, hydration, or heat‐acclimation strategies [35].

Group C supplements such as BCAA, HMB, vitamin E, prebiotics, tyrosine and magnesium are often promoted with mechanistic claims that exceed athlete‐specific evidence. Some may have clinical indications, for example, magnesium in documented deficiency, but routine ergogenic use is not supported. High‐dose antioxidant supplementation may also risk blunting adaptive redox signalling in some training contexts [36].

## 5. The Dark Side of Supplements: Contamination, Mislabelling and Antidoping Risk

Dietary supplements represent one of the most important and often underestimated sources of antidoping risk in modern sport. Products intended to support performance, recovery, or health may expose athletes to medical, legal and professional consequences when manufacturing quality is inadequate or prohibited compounds are present [12–26].

Contamination pathways are multifactorial. Unintentional cross‐contamination may occur when manufacturing lines process multiple products, including products containing prohormones, stimulants, or other pharmacologically active substances. Inadequate cleaning procedures, poor raw‐material control, inaccurate supplier documentation and insufficient batch testing can introduce undeclared compounds. Intentional adulteration is also well documented, particularly in products marketed for rapid weight loss, preworkout stimulation, body composition change, muscle growth, or hormonal enhancement. In these cases, pharmacologically active substances may be deliberately included to produce perceptible effects while being concealed on the label [12–26].

Mislabelling and proprietary blends further impair risk assessment. Ingredients may be listed under unfamiliar chemical names, declared in inaccurate quantities, hidden within blends, or omitted entirely. Some products marketed as ‘natural’ or ‘herbal’ may contain potent bioactive compounds, and online products may originate from jurisdictions with limited quality control. The risk is therefore not binary. A certified single‐ingredient creatine monohydrate product has a substantially different risk profile from an uncertified multi‐ingredient preworkout or ‘testosterone booster’.

Third‐party certification programmes such as Informed Sport, NSF Certified for Sport, BSCG, NZVT and Cologne List reduce the likelihood of contamination by testing selected products or batches against lists of prohibited substances. However, certification is a risk‐minimisation tool, not a guarantee of safety. Certification status can change, logos can be misused, and not all manufactured batches may be tested. Athletes and ASP should verify product and batch status through official databases or applications rather than relying on packaging alone.

Antidoping consequences are governed by strict liability. Even when ingestion is unintentional, the presence of a prohibited substance may trigger an AAF and potentially an ADRV. Intent, fault and source may influence sanctioning, but they do not remove the athlete’s primary responsibility for substances detected in their body [12, 13]. ASP may also face consequences when they administer, recommend, or facilitate prohibited or high‐risk products. This shared responsibility requires structured documentation and clear organisational policies.

## 6. Behavioural Determinants of Supplement Use and Misuse

Supplement use is not determined solely by nutritional need. Athletes often operate in environments where performance pressure, peer norms, coach expectations, sponsorship, body‐image concerns, injury anxiety and social media influence shape decisions [37–41]. Products are frequently promoted using language that suggests safety, naturalness, rapid results, or elite endorsement, all of which may distort risk perception. Athletes may also underestimate the difference between legal availability and antidoping compliance.

Behavioural science is therefore central to inadvertent doping prevention. Educational interventions should not only list prohibited substances but also address common cognitive errors: assuming that ‘natural’ means safe, assuming that a product sold online is compliant, believing that label information is complete, or outsourcing responsibility to a coach, teammate, or influencer. Education should also target ASP, because athletes frequently rely on informal advice from individuals who may not have specialised knowledge of supplement quality control or antidoping regulations.

Effective prevention requires an organisational culture in which supplement decisions are documented, reviewed and normalised as part of athlete healthcare. Teams and federations should establish approved‐product lists, discourage unsupervised online purchases, require batch verification and provide access to qualified sports dietitians or sports physicians. The goal is not to eliminate all supplement use but to make supplement use deliberate, justified and traceable.

## 7. Classification of Supplements According to the ‘Food First but Not Food Only’ Approach

The AIS framework provides a practical classification of supplements into groups according to evidence and appropriateness for use in sport [56]. In the present review, we adapt this framework by explicitly integrating antidoping risk and practical recommendation strength. This is important because efficacy alone does not justify use, and a higher evidence category should not be interpreted as an automatic indication for supplementation.

Group A products may be considered when there is a clear clinical, nutritional, or performance indication and product quality is verified. Group B products require case‐by‐case evaluation because evidence is emerging or context‐dependent. Group C products are generally not recommended for routine performance use, although selected clinical indications may exist. Group D products or product categories should be avoided because they are prohibited, high risk, pharmacologically active, or strongly associated with contamination or adulteration.

Table 2 links supplement categories with practical recommendation strength. These ratings should be interpreted as decision‐support rather than rigid prescribing rules, because individual athlete characteristics, sport demands, nutritional status and product quality may modify the risk–benefit balance.

**TABLE 2:** Evidence‐based classification of dietary supplements adapted from the Australian Institute of Sport (AIS) sports supplement framework and integrated with practical recommendations and antidoping risk.

**AIS-based group**	**Evidence level**	**Examples**	**Primary role**	**ADRV risk**	**Practical recommendation**

Group A	Strong or moderate‐to‐strong evidence in specific scenarios	Carbohydrate products, electrolytes, protein supplements, iron, vitamin D, caffeine, creatine, beta‐alanine, dietary nitrate, sodium bicarbonate	Correction of deficiencies, sports‐food convenience, performance support, training adaptation	Low when certified and appropriate; higher when delivered in multi‐ingredient products	Consider only with a clear indication, individualised plan and verified product quality
Group B	Emerging or developing evidence	Polyphenols, collagen/gelatin + vitamin C, curcumin, omega‐3 fatty acids, probiotics, menthol	Potential recovery, connective tissue support, inflammation modulation, GI or immune support, heat‐perception strategies	Low to moderate; product quality varies	Consider case by case; avoid routine use for unsupported performance claims
Group C	Limited, inconsistent, or low‐quality evidence for performance	Magnesium, BCAA, HMB, vitamin E, prebiotics, tyrosine	No established routine performance benefit in most athletes	Low to moderate depending on product quality	Generally not recommended; use only for specific clinical indications
Group D	Prohibited or high‐risk substances/product categories	Stimulants, SARMs, prohormones, peptide hormones, metabolic modulators, diuretics/masking agents, testosterone boosters	No legitimate role in evidence‐based supplementation	High or very high	Avoid entirely

**Supplement/category**	**Evidence strength**	**SORT/practical strength**	**Most defensible indication**	**Key caution**	

Creatine monohydrate	High	A	Repeated sprint, strength/power, resistance‐training adaptation	Use certified monohydrate; avoid multi‐ingredient blends	
Caffeine	High	A	Endurance, vigilance, reaction time, selected power outcomes	Trial in training; avoid unfamiliar preworkouts	
Carbohydrate/electrolyte sports foods	High	A	Long‐duration, repeated‐session or tournament settings	Individualise to duration, sweat losses and gut tolerance	
Protein supplements	High for supporting protein targets; context‐dependent for performance	A/B	When whole‐food intake is impractical or inadequate	Not needed if dietary intake is sufficient	
Iron	High for correcting deficiency; not for routine use in replete athletes	B	Documented deficiency or low iron availability	Supplement after biochemical assessment	
Vitamin D	Moderate for correcting deficiency; inconsistent performance effects	B	Deficiency/insufficiency, bone health risk	Assess serum status; avoid unnecessary high‐dose use	
Beta‐alanine	Moderate to high	B	High‐intensity efforts ∼1–4 min or repeated high‐intensity work	Requires loading; paraesthesia possible	
Dietary nitrate	Moderate	B	Endurance or intermittent sports in selected responders	Test individually; effects vary by training status and oral microbiome	
Sodium bicarbonate	Moderate	B	High‐intensity repeated efforts	GI tolerance testing is essential	
Collagen/gelatin + vitamin C	Emerging	C	Connective tissue rehabilitation contexts	Use only as an adjunct to loading‐based rehab	
Curcumin/polyphenols	Emerging	C	Recovery or soreness; indirect outcomes	Evidence is heterogeneous; formulation matters	
Omega‐3 fatty acids	Emerging for recovery; stronger for general health contexts	C	Health or inflammation‐related rationale	No routine performance recommendation	
Probiotics	Emerging and strain‐specific	B/C	GI symptoms or illness‐risk reduction in selected contexts	Use strain‐specific evidence	
Menthol	Emerging	C	Heat‐perception strategy	Do not replace cooling, hydration, or heat acclimation	
Magnesium	Low for ergogenic use	C	Deficiency correction only	Avoid routine use without indication	
BCAA	Low when protein intake is adequate	C	Limited role compared with complete protein/EAA	Generally unnecessary	
HMB	Low/inconsistent in trained athletes	C	Possible catabolic or rehab settings	Not routine for performance	
Vitamin E/high‐dose antioxidants	Low for performance; possible adaptation concerns	C	Deficiency or clinical use only	Avoid high‐dose routine use	
Stimulants, SARMs, prohormones, peptide hormones, metabolic modulators and diuretics/masking agents	Not applicable: prohibited/high‐risk	Strong avoidance recommendation	No legitimate supplement role	Avoid entirely	

*Note:* Supplement categories are classified according to the overall strength of evidence, typical indications, estimated risk of antidoping rule violations (ADRVs) and clinical recommendations to support individualised decision‐making within the ‘food first but not food only’ approach.

## 8. Strategies for Risk Management

**FIGURE 1:**
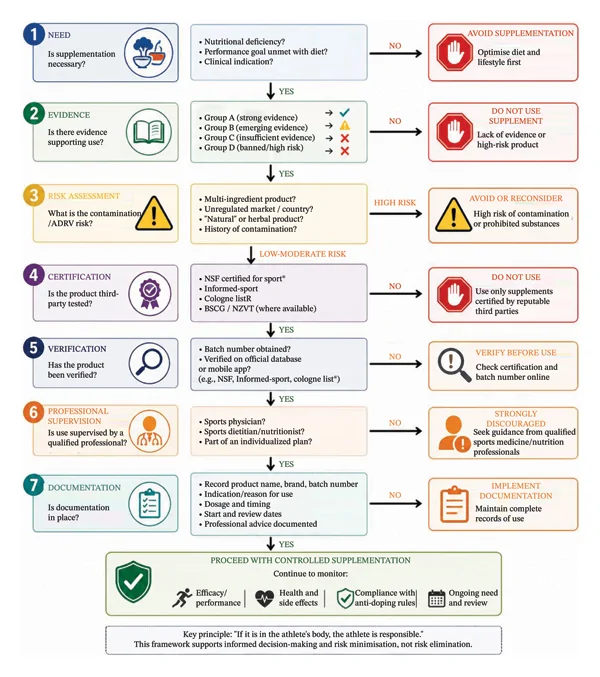
Practical decision‐making framework for supplement use: (1) confirm need; (2) assess evidence and expected benefit; (3) assess athlete‐specific risks; (4) avoid group D/high‐risk categories; (5) select certified products; (6) verify batch and certification status; (7) obtain qualified professional approval; (8) document product and indication; (9) monitor efficacy and adverse effects; (10) review ongoing need. The framework supports risk minimisation, not risk elimination.

Dietary supplementation should be regarded as a controlled, evidence‐informed intervention rather than routine practice [57–61]. Risk management should be continuous rather than limited to a single prepurchase decision, because athlete needs, product formulations, scientific evidence, certification status and antidoping regulations may change over time. We propose 10 practical strategies to reduce the risk of unintentional doping and supplement misuse (Figure 1).

1. Acknowledge the inherent risk. No supplement is entirely risk‐free. Even evidence‐based products may be contaminated, mislabelled, or misused. Risk can be reduced but not eliminated.
2. Prioritise ‘food first’. Dietary intake should be optimised before supplementation. Supplements should address a clear clinical, nutritional or performance need rather than convenience, habit, or marketing pressure.
3. Educate athletes and ASP. Education should address antidoping rules, contamination pathways, misleading marketing, proprietary blends, online purchasing and behavioural drivers of use.
4. Assess necessity and appropriateness. The key question is not whether a supplement can be used, but whether it is necessary for that athlete at that point in training, competition, or recovery.
5. Prefer certified products. When supplementation is justified, use independently tested products. Certification reduces risk but does not guarantee absolute safety.
6. Verify before use. Check certification status and batch number on official databases or applications. Do not rely on packaging or logos alone.
7. Maintain documentation. Record product name, manufacturer, batch number, purchase source, date, dose, duration, indication and professional recommendation. Keep receipts and screenshots where possible.
8. Critically evaluate labels. Avoid products with proprietary blends, unclear ingredients, stimulant‐like compounds, hormone‐related claims, or exaggerated body‐composition promises.
9. Seek qualified guidance. Use sports physicians and sports dietitians with antidoping knowledge. A professional title alone does not guarantee supplement‐safety expertise.
10. Create safe organisational systems. Teams and federations should provide nutrition support, maintain approved‐product policies, discourage unsupervised online purchases and normalise documentation.

## 9. Future Challenges and Research Priorities

Several challenges remain unresolved. First, the appropriate timing and indications for supplementation in adolescent athletes require particular caution. Establishing robust dietary habits, education and performance expectations should generally precede ergogenic supplementation. Second, social media, influencer marketing and online marketplaces are reshaping supplement decision‐making faster than traditional education systems can respond. Third, AI‐generated nutrition recommendations and digital platforms may improve personalisation but may also amplify misinformation if outputs are not evidence‐based, product‐neutral and clinically supervised.

Future research should move beyond efficacy trials alone. Priorities include prospective surveillance of real‐world supplement exposure, standardised contamination testing across product categories, behavioural interventions that reduce risky purchasing, educational programmes for ASP and implementation studies assessing club or federation policies. Research should also address under‐represented populations, including female athletes, adolescent athletes, para‐athletes, masters athletes and athletes outside elite sport systems.

## 10. Conclusions

Dietary supplementation in sport is a complex and potentially high‐risk intervention that extends beyond nutrition into clinical decision‐making, behavioural science, regulatory compliance and ethical responsibility. Selected supplements can provide meaningful benefits when supported by robust evidence, used for a clear indication and sourced from verified products. However, widespread and uncritical use exposes athletes to avoidable risks, including contamination, mislabelling, misinformation and inadvertent ADRVs.

The evidence is not uniform across supplement categories. Creatine and caffeine have relatively strong support for selected performance outcomes, whereas beta‐alanine, nitrate and bicarbonate have more context‐specific evidence. Iron and vitamin D have clearer roles in deficiency correction than in routine performance enhancement. Many recovery‐ or health‐oriented supplements remain supported by emerging or inconsistent evidence. Therefore, supplement decisions should integrate evidence strength, mechanism, athlete characteristics, nutritional need, product quality and antidoping risk.

The proposed framework shifts decision‐making from ‘Does this supplement work?’ to ‘Does the expected benefit outweigh the risk for this athlete, using this product, at this time?’ Reducing inadvertent doping requires education, documentation, third‐party verification, qualified professional oversight and organisational policies that promote risk‐aware supplementation. Such an approach protects athlete health while supporting the integrity of clean sport.

## Author Contributions

L.M., G.L.C., A.J., M.F. and S.P. conceived the article. L.M., G.L.C., V.B., M.V., G.D. and S.P. drafted and revised the manuscript. M.S. and J.P. critically revised the manuscript for important intellectual content.

## Funding

No funding was received for this research.

Open access publishing facilitated by Universita di Pavia, as part of the Wiley ‐ CRUI‐CARE agreement.

## Disclosure

All authors read, edited and approved the final version.

## Consent

The authors have nothing to report.

## Conflicts of Interest

The authors declare no conflicts of interest.

## Supporting Information

Additional supporting information can be found online in the Supporting Information section.

## Supporting information


**Supporting Information** Supporting Table S1. Literature identification and evidence selection process. Supporting Table S2. Evidence appraisal of supplements and supplement‐related substances.
