# Supplementary material for: Reducing the Risk of Unintentional Doping From Supplements: A Practical Guide for Athletes and Support Teams
Source: Transl Sports Med. 2026 Aug 23;2026:5919439. doi: 10.1155/tsm2/5919439 (PMC13501120; doi:10.1155/tsm2/5919439)
Supplement: Supplementary file 1 [file TSM2-2026-5919439-s001.docx]

# Supplementary Material

## Supplementary Table S1. Literature identification and evidence selection process

| **Step** | **Description** |
| --- | --- |
| Identification | PubMed and Scopus searches (January 2000-December 2025) plus manual screening of references from consensus statements, position stands and key reviews. |
| Initial screening | Records considered for relevance to athletes, sports supplements, efficacy, contamination, anti-doping, product certification, behaviour and risk management. |
| Eligibility | Priority given to randomized trials, systematic reviews, meta-analyses, prospective studies, laboratory contamination studies, behavioural studies, consensus statements, position stands and official anti-doping guidance. Excluded: animal-only studies, conference abstracts, non-English papers and articles not directly relevant to sport or supplement safety. |
| Evidence domains | Efficacy and mechanisms; clinical indications; contamination/mislabeling; anti-doping regulation; behavioural determinants; certification/risk management. |
| Narrative synthesis | Evidence interpreted by study design, consistency, biological plausibility, athlete applicability, bias, product quality and anti-doping risk. No quantitative meta-analysis was performed. |

## Supplementary Table S2. Evidence appraisal of supplements and supplement-related substances

| **Supplement/category** | **AIS group** | **Main mechanism** | **Evidence strength** | **Potential role** | **ADRV risk** | **Practical consideration** |
| --- | --- | --- | --- | --- | --- | --- |
| Carbohydrates | A | Exogenous fuel and glycogen availability | High | Endurance, repeated sessions, tournaments | Low if certified | Individualize to event duration and gut tolerance |
| Electrolytes | A | Fluid retention and sodium replacement | High | Heat, prolonged exercise, heavy sweaters | Low if certified | Use sweat-rate/sodium-loss context |
| Protein supplements | A | Essential amino acids and muscle protein synthesis | High | Convenient recovery/adaptation support | Low if certified | Useful when food intake is inadequate or impractical |
| Iron | A | Hemoglobin, oxygen transport, mitochondrial function | High for deficiency correction | Iron deficiency/low ferritin | Low | Biochemical assessment required |
| Vitamin D | A | Bone, muscle and immune-related pathways | Moderate-high for deficiency correction | Deficiency/insufficiency | Low | Assess serum 25(OH)D |
| Caffeine | A | Adenosine antagonism, CNS stimulation | High | Endurance, vigilance, selected power tasks | Low-moderate in blends | Trial dose and timing before competition |
| Creatine monohydrate | A | Phosphocreatine availability and rapid ATP resynthesis | High | Repeated sprint, strength, power | Very low if certified | Use monohydrate; avoid multi-ingredient blends |
| Beta-alanine | A | Muscle carnosine and intracellular buffering | Moderate-high | High-intensity efforts ~1-4 min | Low | Requires chronic loading; paresthesia possible |
| Dietary nitrate | A | Nitrate-nitrite-NO pathway | Moderate | Endurance/intermittent sports in responders | Low | Response varies; test individually |
| Sodium bicarbonate | A | Extracellular buffering | Moderate | Repeated high-intensity efforts | Low | GI tolerance is limiting |
| Collagen/gelatin + vitamin C | B | Collagen synthesis with loading stimulus | Emerging | Tendon/ligament rehab adjunct | Low | Use only with structured loading |
| Curcumin/polyphenols | B | Inflammatory/oxidative signaling | Emerging | Recovery or soreness | Low-moderate | Formulation and dose vary |
| Omega-3 | B | Cell membrane and inflammatory mediator modulation | Emerging | Health/recovery rationale | Low | No routine performance indication |
| Probiotics | B | Microbiota, gut barrier and mucosal immunity | Emerging, strain-specific | GI/immune support | Low | Use strain-specific evidence |
| Menthol | B | TRPM8-mediated cooling perception | Emerging | Heat perception strategy | Low | Does not replace cooling/hydration |
| Magnesium | C | Neuromuscular cofactor | Low for ergogenic use | Deficiency correction only | Low | Avoid routine use without indication |
| BCAA | C | Leucine-related anabolic signaling | Low when protein adequate | Limited role | Low | Inferior to complete protein/EAA |
| HMB | C | Leucine metabolite; reduced protein breakdown | Low/inconsistent | Possible catabolic contexts | Low | Not routine in trained athletes |
| Vitamin E/high-dose antioxidants | C | Antioxidant activity | Low for performance | Deficiency/clinical use | Low | May blunt adaptation at high doses |
| Prebiotics | C | Microbiota substrate | Low | Gut health rationale | Low | Limited performance evidence |
| Tyrosine | C | Catecholamine precursor | Low | Cognitive stress contexts | Low-moderate in blends | Not routine |
| Stimulants (DMAA/ephedrine-like) | D | CNS stimulation | Not applicable | No legitimate supplement role | High | Avoid |
| SARMs | D | Androgen receptor modulation | Not applicable | No legitimate supplement role | Very high | Avoid |
| Prohormones/testosterone boosters | D | Hormonal modulation | Not applicable | No legitimate supplement role | Very high | Avoid |
| Peptide hormones/GH secretagogues | D | Endocrine stimulation | Not applicable | No legitimate supplement role | Very high | Avoid |
| Metabolic modulators | D | Altered substrate metabolism | Not applicable | No legitimate supplement role | Very high | Avoid |
| Diuretics/masking agents | D | Urinary dilution or detection interference | Not applicable | No legitimate supplement role | Very high | Avoid |
